# Increasing Latinx representation in the US medical schools: A top-ranked medical school's experience

**DOI:** 10.3389/fpubh.2022.907573

**Published:** 2022-09-16

**Authors:** Leonor Corsino, Félix M. Chinea, Linton Yee, Anthony T. Fuller

**Affiliations:** ^1^Department of Medicine, Division of Endocrinology, Metabolism, and Nutrition, Duke University School of Medicine, Durham, NC, United States; ^2^Duke University School of Medicine, Durham, NC, United States; ^3^Duke University School of Medicine, Latino Medical Student Association, Durham, NC, United States; ^4^Health Equity and Inclusion Strategy, Doximity, San Francisco, CA, United States; ^5^Department of Pediatrics, Duke University School of Medicine, Durham, NC, United States; ^6^Department of Neurosurgery, Division of Global Neurosurgery and Neurology, Duke University School of Medicine, Durham, NC, United States; ^7^Duke Clinical and Translational Science Institute, Center for Pathway Programs, Duke University School of Medicine, Durham, NC, United States

**Keywords:** Latino, Hispanic, Latinx, workforce, recruitment, enrollment

## Abstract

Despite the increasing racial and ethnic diversity of the general US population, many, if not most, medical schools fall short of matriculating students reflective of this change in diversity. The Latinx community constitutes nearly 20% of the US population and is expected to rise substantially in the coming decades. Over the past 20 years, the number of Latinx students applying to and being admitted to medical school has grown but remains below 4% of the total medical student body composition. Several factors contribute to the under-representation of Latinx students in medical schools that include access to secondary education, finances, lack of mentorship or advice, and a multitude of other structural inequities and system-level biases. Diversity, equity, and inclusion are often named as key pillars of workforce development across the US medical schools and academic institutions. Despite significant efforts, medical schools continue to have low Latinx representation within their student body, and recruitment efforts often lack sustainability. In this manuscript, we share our experience of increasing Latinx student representation within a top-ranked medical school in the US southeast region. We have discussed the barriers we faced in enrolling and attracting Latinx students' amidst similar under-representation of Latinx faculty, staff, and leadership and the challenges of financial support for applicants and financial aid packages for admitted students. The strategies we implemented to achieve an increased representation of Latinx students in the School of Medicine (SOM) included revitalizing the Latino Medical Student Association (LMSA), ensuring Latinx student representation within the SOM admission committee that included Latinx faculty as active participants in recruitment and retention efforts, redesigning the medical Spanish course, and creating dedicated outreach plans during second look weekend for interested Latinx students and active outreach to applicants and accepted students by current students. In combination, these efforts led to a significant increase in Latinx representation in the SOM student body from 2.6% in 2009 to 12.2% in 2021. We will conclude by discussing our ongoing challenges and our approach to sustain and improve Latinx representation in our medical school.

## Introduction

The diversity of the healthcare workforce remains a challenge in the United States, especially in medical schools (1). Despite the increase in diversity in the US general population, diversity in medical schools remains an unmet challenge. In recent decades, the number of Latinx (i.e., a term centering on both racial and gender equity that is inclusive of those who identify as Hispanic, Latino, Latina, or Latine) students applying and admitted to medical school has increased, however (1), the number remains significantly lower than the proportional representation within the US, 18.5% (2). According to recent US census data, the Latinx population is expected to increase significantly and will represent about 28% of the country by 2060 (3). Medical school matriculates are 6.2% (4). Several factors have been cited as contributing to the under-representation of Latinx students in medical schools that include structural inequities, limited access to education, finances, and lack of mentorship or advice (5–7).

Our school has been recognized for its efforts to increase diversity in the healthcare workforce and for the success of matriculating one of the most diverse medical school classes in the country (8). Efforts to intentionally diversify our study body started decades ago with work led by our late colleague Dr. Brenda Armstrong. During her tenure, as Dean of Admissions for the School of Medicine (SOM), our school increased Black, Indigenous, and people of color (BIPOC) students. Representation of Latinx students remained a challenge. In 2009, a Latinx student (Chinea) under the mentorship of (Corsino) revitalized the Latino Medical Student Association (LMSA) chapter at the Duke SOM with one of the main goals to increase Latinx representation in the school. In this manuscript, we have shared strategies implemented to achieve an increased representation of Latinx students in the SOM and our success to date.

## Strategies and their implementation

### Strategies

Several strategies (Table 1) have been implemented since 2009 to increase the Latinx enrollment within our school. These have been developed and led by the school's LMSA chapter leadership with support from the Multicultural Resource Center (MRC), the Office of Diversity and Inclusion (ODI), the Department of Medicine, and the Medical School Admissions Office and their leadership. The approach utilized to develop these strategies started with the recognition of the lack of representation.

**Table 1:** Strategies implemented to increase Latinx representation at the medical school.

**Strategy**	**Approach**
Revitalization of the Latino Medical Student Association	Needs assessment, identification of resources within the school, leadership, recruitment of faculty advisor, recruitment of students to serve as leaders and members.
Increase representation at the local, regional, and national level	Collaboration with other local LMSA chapters, representation at the local, regional, and national LMSA meetings.
Increase representation and voice at the SOM	Collaboration with faculty departments in sponsorship of Ground Rounds and larger events e.g., Department of medicine Ground Rounds, screening of the HBO documentary Clinical de Migrantes, sponsoring with Office of Diversity and Inclusion, the Building the Next Generation of Academic Physicians (BNGAP) Meeting.
Incorporate the Latinx voice in the school admissions committee	One LMSA representative joins the admission committee each year since 2014.
Active recruitment	LMSA recruitment committee emails and calls accepted Latinx students Sponsored social events during the second-look weekend.
Redesigned the SOM medical Spanish course	Deploy survey to determine students perception of the need and the existing course Utilize data from the survey to redesign the course to fit students' needs Presented the course to the curriculum committee Designed with 3 components: individual learning (40 h. of modules completed at home), Clinical Knowledge sessions (16 h sessions led by Spanish-speaking faculty); Integral clinical experience (students can practice their history-taking skills in Spanish with standardized patients).
Build community	Sponsored social gatherings and events including café y conversación, volunteer at vaccination and COVID testing events, celebrate el Día de los Muertos with movie and ofrenda (altar), welcoming events for new students, etc.

**Figure 1:**
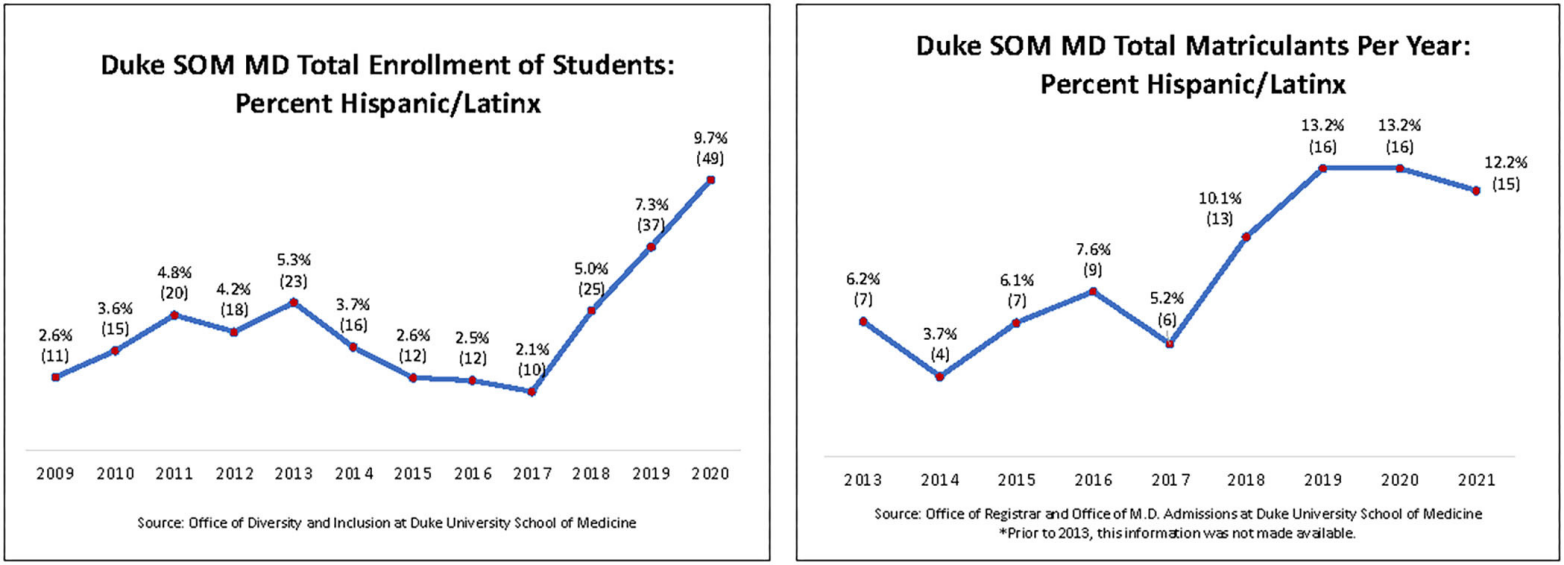
The left panel shows the percentage of Hispanic/Latinx students enrolled at Duke SOM from 2009 to 2020 as a function of total enrollment. The right panel shows the percentage of Hispanic/Latinx student matriculants per year at Duke SOM from 2013 to 2021 as a function of total matriculants.

At the time, there were only 2.6% of self-identified Latinx students enrolled in the school (Figure 1). Of those, only a few were engaged or interested in Latinx-related issues. One student assumed the role of leader (Chinea) and was strongly committed to these efforts. The student identified a faculty member (Corsino) from the small number of existing Latinx faculty at the time to serve as faculty advisor for a Latinx-focused student interest group, LMSA. Together, they started working toward recruiting and incentivizing other students from all years to join LMSA. The initial efforts, identifying a student and faculty led to revitalized LMSA and recruitment of other students to join the LMSA, helped with the establishment of the LMSA chapter that included a small cohort of students. This group developed short- and long-term plans to increase recruitment to LMSA and the school, increase activities to foster and establish community, and amplify Latinx students' voices within the school, locally, and nationally.

Due to the small number of students actively engaged in LMSA, students intentionally collaborated with other local and regional LMSA chapters to organize events to promote the work of their chapters and to mentor and advise Latinx students pursuing a career in medicine. A student and/or the faculty advisor typically joined LMSA national meetings to represent the school chapters, recruit more applicants, and share more about the school. The chapter sponsored several events that included medical Spanish interviews, social events, and ground rounds with a Latinx leader in academic medicine (co-sponsored by the Department of Medicine). These initial efforts were successful to bring more applicants to our school. It was clear, however, that more intentional efforts needed to be implemented to increase the number of Latinx students applying and matriculating in our school.

In 2014, LMSA approached the school admission office and the Dean of Admissions with the request to add an LMSA student representative to the admissions' committee. The goal was to have the Latinx voice represented in the applicant's review. The first representative of LMSA in admissions started for the 2015–2016 academic year. Dr. Fuller, the co-author, was the first student representative in the admissions committee. Including a student in the admissions committee provided the much-needed voice that successfully increased the number of Latinx students offered acceptance. A persistent challenge faced by the school was that while the numbers of offers were increasing, the numbers of offers accepted were low. LMSA in collaboration with others within the school implemented new strategies.

### New strategies

The Latino Medical Student Association students developed a plan to reach out to all self-identified Latinx students accepted to the school *via* email or phone. The purpose of the email or phone call was to share more information about the Duke School of Medicine LMSA chapter, the life of Latinx students at the school, and share data regarding the existing demographic diversity of Durham, NC and the existing opportunities to work with the local Latinx patient population. This outreach offered an opportunity to answer candidates' questions and to provide them with information regarding the Latinx student experience in our school. These efforts have been significantly successful, currently, the number of students engaged in LMSA is 60 students in the year 2022, and the number of matriculated students is 12.2% in 2021 (Figure 1).

The increase in numbers achieved during the last 13 years has been significant. The active engagement, advocacy, and leadership of the school LMSA chapter have also been significant and impactful. In addition, to the increase in Latinx student representation, the chapter expanded its reach and efforts to collaborate with other departments to sponsor grand rounds in other specialties (e.g., Neurosurgery), showcasing successful and accomplished Latinx faculty from around the country to provide the students with exposure to faculty that looks like them, supported larger events with national leaders, such as the screening of the Home Box Office (HBO) documentary, Clinica de Migrantes, collaborated with the ODI for the Building the Next Generation of Academic Physician (BNGAP) event, developed the Scholarly Academy for Latinxs United for Diversity (SALUD) program, https://sites.duke.edu/salud/, and redesigned the Medical Spanish course at the School of Medicine, called Advanced Clinically Centered Education In Spanish (ACCES) (Table 1). The newly redesigned Medical Spanish course targets first-year medical students with an intermediate to and advance level of Spanish. The new course, developed by students, for students, and with guidance from experienced medical Spanish faculty and educators, aims to train providers to confidently communicate and care for Spanish-speaking patients. The increasing number of the Latinx population in the US increased the need to train more providers to care for this segment of the population. The offering of this course serves as a strong attractive offering for current and prospective students.

## In summary

Diversifying the health professional workforce, particularly physicians, remains a challenge. Although progress has been made in recent years, the under-representation of Latinx students remains low as compared to the current and projected representation of the US Latinx population. Efforts to increase the number of applicants and matriculating number of accepted and graduating students require a multifaceted and multidisciplinary approach. Student organizations, with the support from school leadership, can make a significant impact in achieving progress. Ongoing challenges include the existing structural inequities and system-level biases that limit Latinx students from pursuing and successfully completing medical school.

## Data availability statement

The original contributions presented in the study are included in the article/supplementary material, further inquiries can be directed to the corresponding authors.

## Author contributions

LC: gather the data, develop the manuscript idea, wrote, and edit the manuscript. FC, AF, and LY: edit and manuscript preparation. All authors contributed to the article and approved the submitted version.

## Conflict of interest

The authors declare that the research was conducted in the absence of any commercial or financial relationships that could be construed as a potential conflict of interest.

## Publisher's note

All claims expressed in this article are solely those of the authors and do not necessarily represent those of their affiliated organizations, or those of the publisher, the editors and the reviewers. Any product that may be evaluated in this article, or claim that may be made by its manufacturer, is not guaranteed or endorsed by the publisher.
